# Clear effects on root system architecture of winter wheat cultivars (*Triticum aestivum* L.) from cultivation environment and practices

**DOI:** 10.1038/s41598-024-61765-1

**Published:** 2024-05-15

**Authors:** Jonathan E. Cope, Fede Berckx, Johan Lundmark, Tina Henriksson, Ida Karlsson, Martin Weih

**Affiliations:** 1 Department of Crop Production Ecology, Swedish University of Agricultural Sciences, 750 07 Uppsala, Sweden; 2 Lantmännen Lantbruk, Udda Lundkvists väg 11, S-26881 Svalöv, Sweden; 3 Department of Immunology, Genetics and Pathology, Clinical Genomics Uppsala, Science for Life Laboratory, Uppsala University, 751 85 Uppsala, Sweden

**Keywords:** Root system architecture, Root growth, *Triticum aestivum*, Precrop effect, Wheat yield, Plant sciences, Plant development, Agroecology

## Abstract

Roots play a pivotal role in the adaption of a plant to its environment, with different root traits adapting the plant to different stresses. The environment affects the Root System Architecture (RSA), but the genetic factors determine to what extent, and whether stress brought about by extreme environmental conditions is detrimental to a specific crop. This study aimed to identify differences in winter wheat RSA caused by cultivation region and practice, in the form of preceding crop (precrop), and to identify if modern cultivars used in Sweden differ in their reaction to these environments. This was undertaken using high-throughput phenotyping to assess the RSA. Clear differences in the RSA were observed between the Swedish cultivation regions, precrop treatments, and interaction of these conditions with each other and the genetics. Julius showed a large difference between cultivars, with 9.3–17.1% fewer and 12–20% narrower seminal roots. Standardized yield decreased when grown after wheat, 23% less compared to oilseed rape (OSR), and when grown in the Southern region, 14% less than the Central region. Additionally, correlations were shown between the root number, angle, and grain yield, with different root types being correlated depending on the precrop. Cultivars on the Swedish market show differences that can be adapted to the region-precrop combinations. The differences in precrop effect on RSA between regions show global implications and a need for further assessment. Correlations between RSA and yield, based on root-type × precrop, indicate different needs of the RSA depending on the management practices and show the potential for improving crop yield through targeting genotypic and environmental conditions in a holistic manner. Understanding this RSA variance, and the mechanisms of conditional response, will allow targeted cultivar breeding for specific environments, increasing plant health and food security.

## Introduction

Wheat is an important part of Swedish agriculture, with approximately 3.2 million tonnes of wheat being produced in 2020, accounting for 5.1 billion SEK (€486 million). Sweden extends 1572 km from north (69°N) to south (55 °N) and thus the agricultural, climatic, and soil conditions are very different^1^. Between diverse regions, and due to differing agricultural practices, the growth conditions and environments differ greatly, and the suitability of a cultivar will largely be related to the adaptation to these factors. Adaptation to differential climates and soil conditions involves a range of different elements, and the root system is an important component in optimising a plant to these conditions or environments^2^. Likewise, different farm practices can improve the environment for root growth^3^. Certain root morphological characteristics adapt wheat to best utilize resources in different environmental conditions with abiotic stresses such as water-limited^4^, and nutrient-limited soils^5^. Additionally, root morphology has been shown to allow crops, such as maize, to limit the disease development of soil-borne pathogens^6^.

Root system architecture (RSA) is the collective term for the morphological features of the root system, including root number, angle, and length. There are two main groups of roots in cereal crops such as wheat: seminal roots emerging from the seed, and nodal roots emerging from the stem tissue (including both crown and brace roots)^7^. The system of RSA development is highly plastic and allows for the adaptation to environmental changes^8^, which, in turn, can maintain yield in different conditions by adapting to different stresses. This is of particular importance in light of the changing climate and the challenges to crop production that will occur with this^9^. Despite this strong plasticity in reaction to the environment, the architecture and its response are largely controlled by genetic factors^10^, and how this genetic factor expresses itself depends on the environment (Genotype × Environment (G × E) interaction). Modern cereal cultivars have reduced the variation in the RSA compared to landraces^11^. These newer cultivations have a deeper, but less branched, root system that allows a denser sowing rate, and thus increases yield in an agricultural scenario^12^. However, large variation in root traits still exists within the modern populations of Europe, and the rest of the world^13^.

The plasticity of RSA plays an important role in the adaptation of a crop to environmental conditions. Higher yields have been linked to certain RSA traits, such as seminal root number in wheat^14^. Root number has also been shown to be positively correlated with both yield under low moisture environments^4^ and drought tolerances^15^. Narrow root growth angles promote deeper root growth and are linked with improved access to water and nutrients under drought stress^16^. Shallower roots have been linked with the ability to produce adventitious surface roots quickly, this provides flooding tolerance in maize crosses^17^ and other grasses^18^. Nutrient acquisition is also affected by the RSA, affecting environmental adaptation. Increased seminal root^19^ and crown root number^20^ in maize have been linked with increased nutrient acquisition. This is supported by the work of Liu, et al.^21^, which has shown thinner and shallower roots to be associated with increased N uptake efficiency in wheat. However, Schneider, et al.^22^ showed that maize with fewer, but thicker, nodal roots exhibited better growth under nitrogen-limiting conditions. Alternative RSA can thus play a role in adapting crops to different environments.

Additionally, farm management practices will also affect the growing environment. One such practice that is commonly used is rotational cropping. Rotational cropping is the act of switching which crop is grown on a specific piece of land between seasons^23^. This changes the environment as the preceding crop (precrop) will affect soil conditions such as biopores^24^, weed and pathogen burden^25^, resource availability^26^, and microbial community^27^. Rotational cropping has been shown to increase yield in crops^26,28^. Most work in understanding the effect of different precrops on the RSA is understanding the soil structure after a crop, such as biopores, and thus studies on other aspects of RSA are limited^24^. There are still gaps in the understanding of the effects different precrops and associated ecosystem services have on the RSA^29^. RSA holds much potential in breeding crops that are adapted to a specific environment. Filling the gap in this knowledge will unlock the use of the large diversity of RSA in the breeding population^13^.

Assessment of RSA with relation to plant health and growth is often a high-cost or low-throughput method, resulting in limited implementation into breeding programs^30^. Subsequently, the main changes in RSA through breeding have been untargeted^11^. The principle method used to assess RSA in this study was a high-throughput phenotyping technique called ‘shovelomics’^31^. Shovelomics is a high throughput screening of plants from the field by digging the root core and removing the bulk soil, which has been used to assess the differences in basic RSA between different cultivars of cereal crops^31^, including wheat^32^.

The main objective of the present study was to detect how the RSA of modern winter wheat cultivars is affected by three factors: region, pre-crop, and cultivar. We also looked at their interaction effect. Moreover, the study assessed how these RSA traits are expressed over multiple growing seasons, and how these factors relate to yield. To assess the effect of precrop on the RSA over different regions of Sweden systematically would require large amounts of resources, to establish field trials and collect samples. Therefore we partnered with Lantmännen Lantbruk (Svalöv, Sweden) to collect samples from already established field trials. These field trials consist of two connected trials (one with a conventional precrop, one with a wheat precrop), replicated in two locations throughout Sweden (Skåne and Västra Götaland). Measurements of the RSA were taken at three time points over two years using the shovelomics method^31^. The results from this study will allow us to form hypotheses about the effect of precrop on the RSA, and therefore the role of rotational cropping choice in adapting the root system for better yield and yield stability.

## Results

### Differences between years

The RSA showed differences between the years (Supplementary Fig. 1), including nodal root number and angle (p-values < 0.001), predominantly due to the increase in the former at flowering in 2021 (Supplementary Fig. 1A), and seminal root number (p = 0.027), but not seminal root angle (p = 0.214; Supplementary Fig. 1D).

### Effect of region

**Figure 1:**
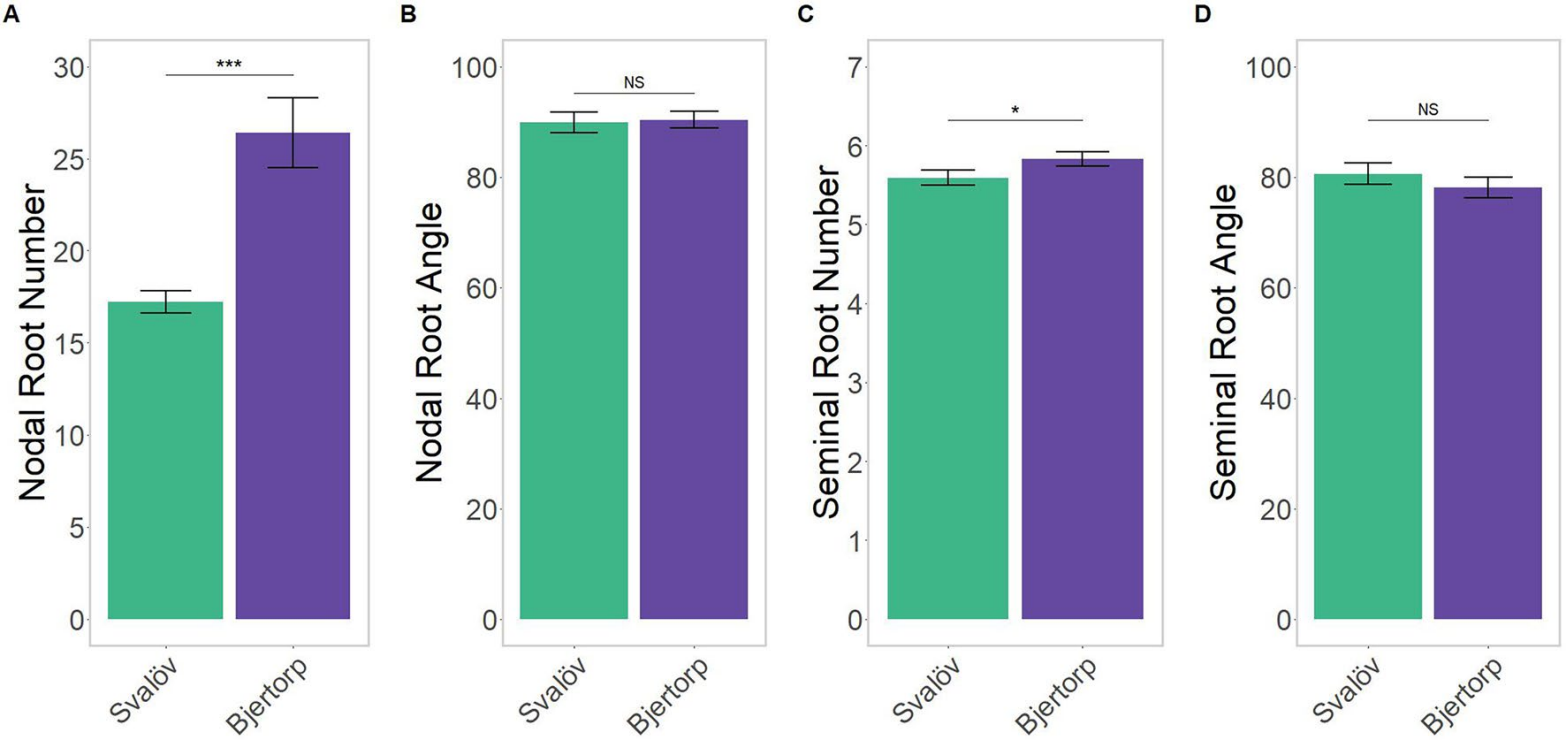
Differences in nodal root number (**A**) and angle (**B**), and seminal root number (**C**) and angle (**D**) between the two growing regions of Sweden–Svalöv in Skåne (green) and Bjertorp in Västergötland (purple). The significance is based on the estimated marginal means from the emmeans package in ‘R’^70^; denotations are ‘*’ for p < 0.05, ‘***’ for p < 0.0001, and ‘NS’ for no significance.

The main differences caused by region are in the nodal root number (p = 0.005; Fig. 1A) with an approximate average of 50% more nodal roots when grown in Bjertorp compared to Svalöv. Less evident, but still significant (p = 0.044), is the small increase in the seminal root number when grown in Bjertorp compared to Svalöv (Fig. 1C). This increased root number had no effect on the average root angle between regions for both nodal and seminal roots (Fig. 1B, D, respectively), with the former showing near identical average angles between sites.

**Figure 2:**
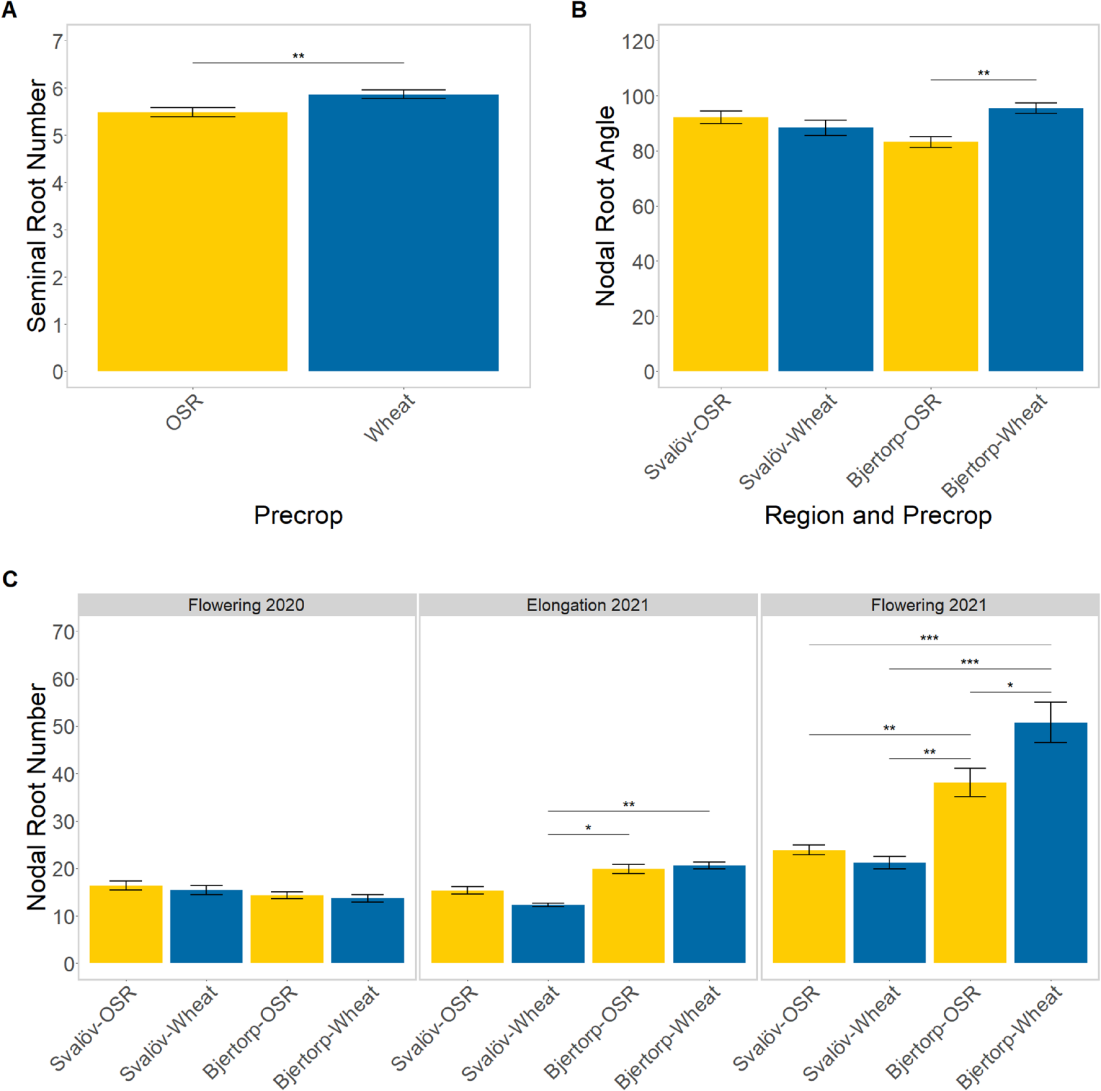
Differences in nodal root number (**A**), nodal root angle (**B**), and seminal root number (**C**), between the two different preceding crops–OSR (yellow) and wheat (blue). The nodal root data (**A**,**B**) is further split between the two different growing regions in Central (Bjertorp) and Southern Sweden (Svalöv) each with two field sites for the different precrops. The nodal root number (**A**) is further faceted by the sampling period. The significant difference is based on the estimated marginal means from the emmeans package in ‘R’^70^, and is only within the same sampling period for nodal root number (**A**). The denotations are ‘*’ for p < 0.05, ‘*’ for p < 0.01, and ‘***’ for p < 0.0001. Faceted data for seminal root number and angle is displayed in Supplementary Fig. 7.

Significant differences were also found in the interactions between the sampling date and region for nodal root number (p = 0.001) and angle (p < 0.001). This difference can be seen as the large increase in nodal roots at the flowering stage in the second year of the trials at Bjertorp (Fig. 2A). Regional differences in nodal root number changed depending on the year, with Bjertorp only having significantly more roots in the second year.

### Effect of the preceding crop

The effect of the precrop by itself showed a significant difference in only the seminal root number (p = 0.008). However, differences in the interaction of precrop were seen, with significant differences in nodal root number (p = 0.042) and angle (p = 0.002) in the region-precrop interaction and the latter in combination with sampling (p = 0.023). No significant precrop effect was seen in the seminal root angle.

The effect of the differing precrops mainly showed region and year-dependent differences. In Bjertorp, no differences between precrops could be seen, except for during flowering in the second year, where there were a lot more nodal roots after a wheat precrop (p = 0.042; Fig. 2A).

A conflicting pattern showed the nodal root angle was affected by different precrops depending on region (p = 0.0018). Whilst differences in precrop effect were small, it was clearly seen in the Bjertorp trials that wheat after wheat caused a larger nodal rooting angle compared to an OSR precrop (Fig. 2B).

Significant differences (p = 0.008) in the seminal root number were also seen between precrops (Fig. 2C), with an increase in seminal root number when sown after a wheat crop. Whilst this remained consistent in both regions, the post hoc test showed no significant difference when comparing within each region.

### Effect of cultivar

#### Seminal roots

**Figure 3:**
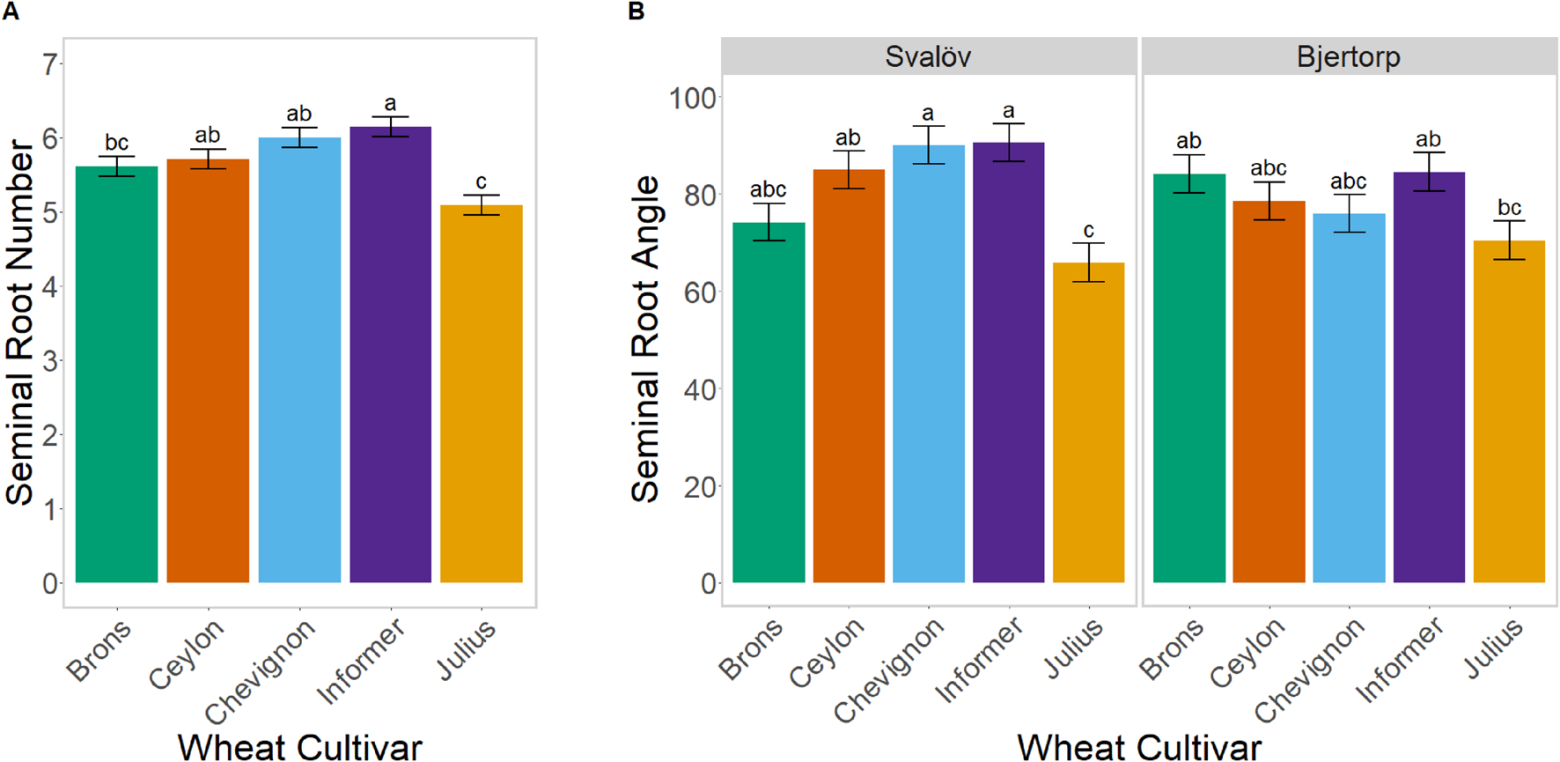
Differences in seminal root number (**A**) and angle (**B**), between different cultivars (coloured) of wheat plants field-grown in two growing regions in Central (Bjertorp) and Southern Sweden (Svalöv)—the data is divided by these regions for seminal root angle (**B**). The significance is based on the estimated marginal means from the emmeans package in ‘R’^70^; cultivars without a matching letter, are significantly different (p < 0.05).

The major difference in both seminal root number and angle (p-values < 0.001) is primarily caused by the difference seen in the cultivar Julius. Brons and Ceylon had slightly fewer seminal roots than Informer and Chevignon, but Julius had much fewer, approximately 9.3–17.1%, than all other cultivars (Fig. 3A).

Overall Julius had a significantly narrower seminal rooting angle from the other cultivars (Fig. 3B), approximately 12–20% less than the other cultivars–this was seen in both fields in all samplings, except for Bjertorp in 2020 (Supplementary Fig. 2). The cultivars also had significantly different reactions to the different regions (p = 0.018), with some cultivars having smaller rooting angles in Svalöv, with other cultivars having smaller rooting angles in Bjertorp (Fig. 3B).

#### Nodal roots

**Figure 4:**
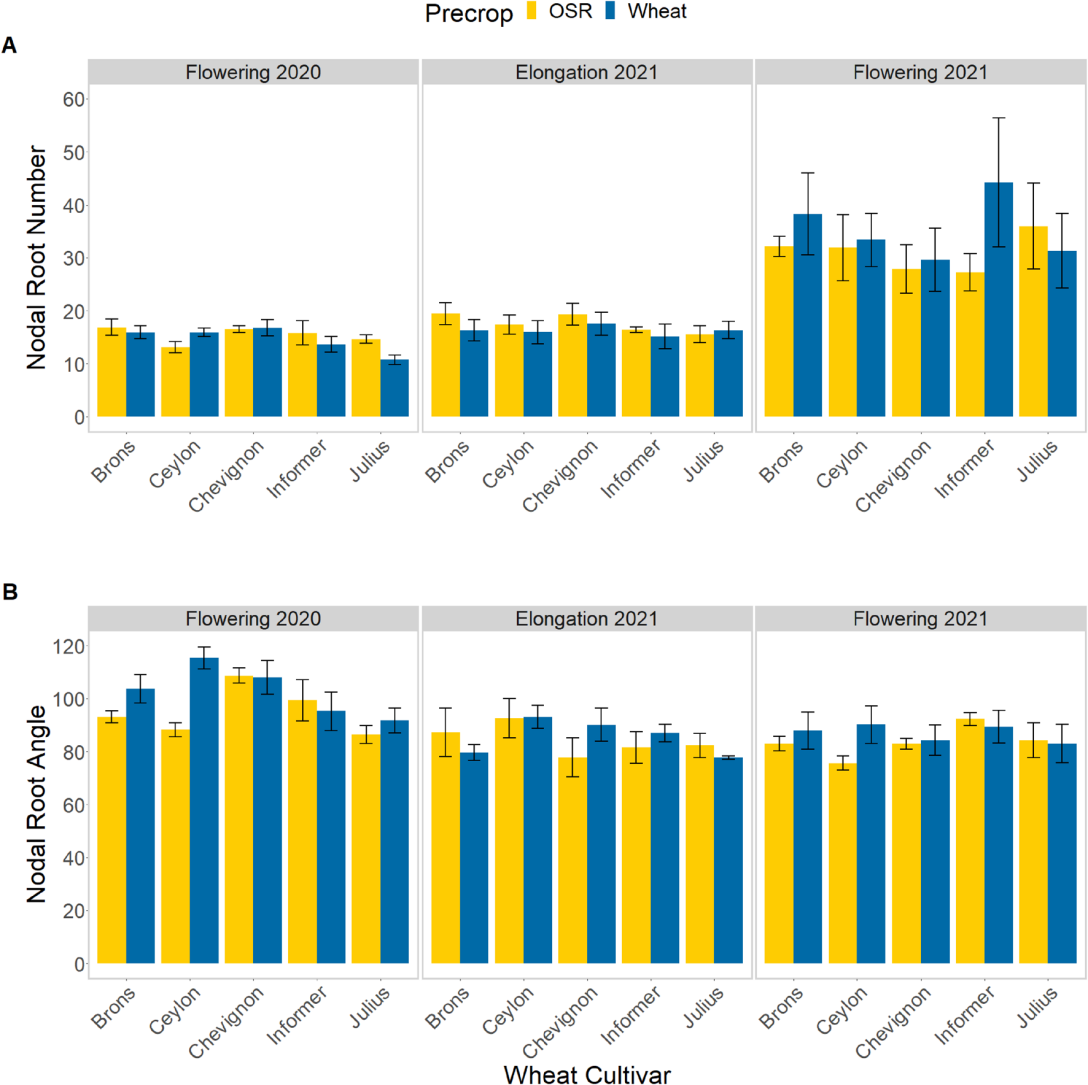
Differences in nodal root number (**A**) and angle (**B**), between different cultivars of wheat plants field-grown in two growing regions in Central and Southern Sweden. The data is divided by sampling period, and coloured depending on precrop–OSR (yellow) or wheat (blue). Pairwise comparisons are noted in Supplementary Table 1.

The significant difference in the nodal root number was dependent on the cultivar and sampling date (p = 0.003; Fig. 4A). The significant difference (p = 0.006) in nodal root angle was largely seen in the cultivar Ceylon during flowering, which showed an approximate 19% increase (becoming shallower) when grown after a wheat precrop, compared to after OSR (Fig. 4B; p = 0.026); but not seen during elongation.

#### Grain yield

Differences in the standardised yield were shown to be significant within the region, precrop, and harvest year (p-values < 0.001), as well as many of the interactions. Large decreases in yield (23%) were shown when grown after wheat, compared to OSR, and lower yields (14%) in Svalöv over Bjertorp (Supplementary Fig. 3A). This differed between the years, with smaller decreases in yield after wheat, and no average difference between regions, in 2020 (Supplementary Fig. 3B). Differences in yield between cultivars were smaller, with Chevignon and Informer producing the most grain, significantly more than the lowest producer, Ceylon (Supplementary Fig. 3C).

**Figure 5:**
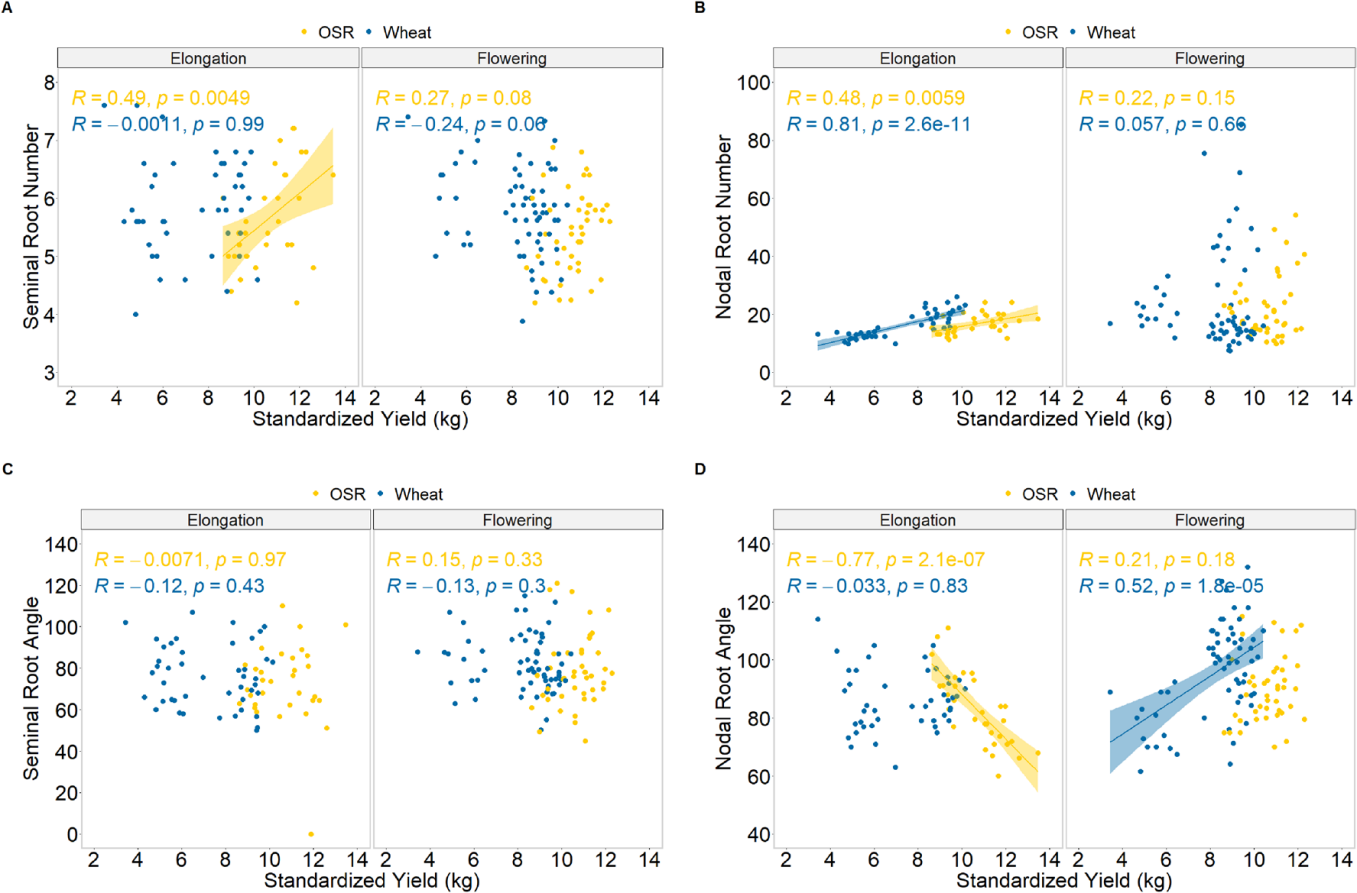
Pearson’s correlations of yield values, standardized to account for differing moisture levels, with seminal (**A**,**C**) and nodal (**B**,**D**) root numbers (**A**,**B**) and angles (**C**,**D**) of wheat plants field-grown in two growing regions in Central and Southern Sweden. Regression lines are fitted based on the precrop of either OSR (yellow) or wheat (blue), with a facet grid dividing the root data by collection during the elongation phase (2021) and flowering phase (2020 & 2021).

Positive correlations were found between the root number and the standardised yield, dividing the samples based on the different precrops. In the seminal roots, this was only exhibited after an OSR precrop during elongation (R = 0.49, p = 0.005; Fig. 5A) and during flowering (R = 0.32, p = 0.034; Spearman rank correlation not illustrated). Strong positive correlations were also seen in the nodal root number during the elongation phase (Fig. 5B) with both an OSR (R = 0.48, p = 0.006) and wheat (R = 0.81, p < 0.001) precrops.

Correlations between standardized yield and root angle were only seen in the nodal roots, with differing patterns based on the preceding crop, and no significant correlation was seen between seminal root angle and yield at either stage (Fig. 5C). Only when the precrop was wheat, did increased standardized yield correlate with a wider nodal root system during the flowering stage (R = 0.52, p < 0.001; Fig. 5D). Conversely, when the precrop was OSR, a wider nodal root number during the elongation phase correlated to a decrease in yield (R = 0.77, p < 0.001; Fig. 5D). This pattern is further reflected in the average variety data, in part, with negative rank correlations between nodal root angle and standardized yields (Supplementary Fig. 4A). However, rank correlations showed no significance in the varieties’ average in the flowering stage after a wheat precrop (Supplementary Fig. 4B).

**Figure 6:**
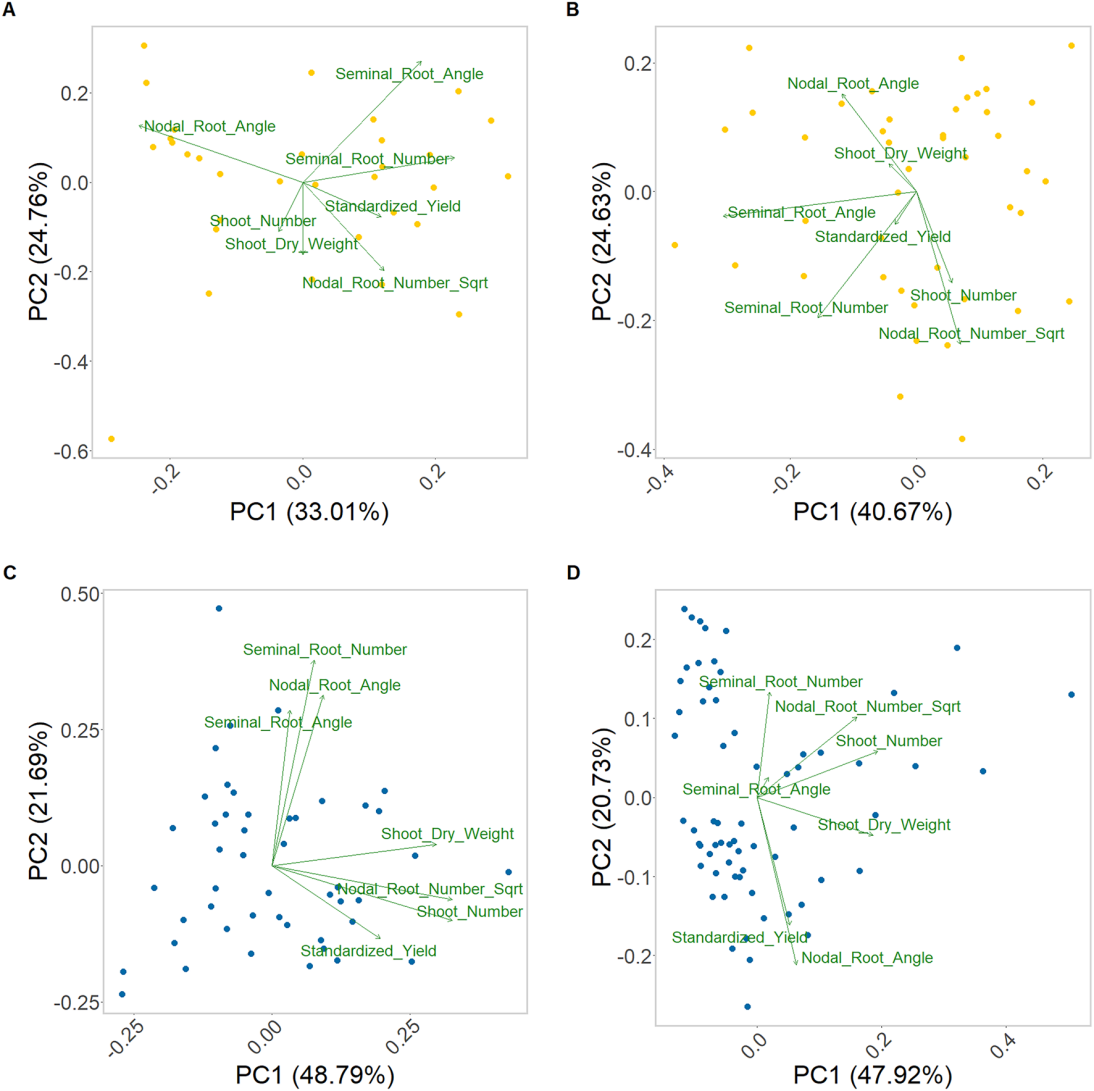
Principle component analysis of shoot characteristic, root system architecture, and yield values, standardized to account for differing moisture levels of winter wheat plants field-grown in two growing regions in Central and Southern Sweden. The values are standardized to the mean of the entire Elongation (**A**,**C**) or Flowering (**B**,**D**) datasets and then split based on the precrop of either OSR (**A**,**B**; yellow) or wheat (**C**,**D**; blue).

Principle component analyses complement these results. With seminal root number showing a positive alignment in both elongation and flowering when grown after an OSR crop (Fig. 6A, B, respectively), whereas this is not seen when grown after wheat, where they are either orthogonal, at elongation (Fig. 6C), or negatively aligned during flowering (Fig. 6D). Additionally, we also see that during elongation the nodal root angle is negatively aligned after OSR (Fig. 6A), but orthogonal after wheat (Fig. 6C), whilst during flowering it is orthogonal after OSR (Fig. 6B) and positively aligned after wheat (Fig. 6D).

## Discussion

The main strength of this study is the consideration of the regional, management, temporal, and genetic effects in conjunction with each other, and their interaction, on root characteristics along with crop yield. We are aware that this integrated perspective has its drawbacks due to the complexity of the interactions involved, along with other factors that cannot be systematically separated—such as non-precrop farm management differences between sites. Despite the drawbacks, we still believe that our results illustrate the relevance of all investigated factors for the assessment of RSA. Thus, we identified complex differences in RSA based on the genetic-environment interactions and links between root traits and yield in certain environments, as well as cultivar-specific sensitivities to distinct stresses in the region/management.

### RSA variability across regions and precrops

This study has shown that RSA will significantly differ between the different soils within Sweden. Other studies have similarly shown significant differences in RSA that have been grown in contrasting soil types and structures^33^, compounded with environmental effects, which have shown to also affect RSA^34^ due to stimuli and pressures within the soil from both biotic and abiotic sources^35^. Additionally, there are interaction effects between region and precrop, with different regions causing the effect of the precrop to be reduced, enhanced, or reversed. The latter is shown in the nodal root number which showed different trends in precrop effects in Svalöv compared to Bjertorp. This difference in precrop effect depending on the region has been noted in the yield in grain legumes^23^, with soil-type-dependant differences between precrops being seen in the phosphorus uptake in wheat^36^, and thus has implications on a global scale. The differences seen in these studies could, in part, be explained by our results, as we have shown that the environment can alter the precrop effect on RSA–changing how the root system is organised, which would not be seen in the root biomass assessment done in Wang, et al.^36^. Differences in the weather should be factored in (Supplementary Fig. 5), as they are inexorably connected to regional and temporal differences.

The full extent of the impact of soil type is still being uncovered, and the effects precrops have on RSA are largely underexplored outside the area of biopores^37^, which do not remain in the surface soil after tilling^38^. Different precrops have also been shown to affect the fungal root symbionts of the following crop^39^, the redistribution of soil nutrients from deeper soil layers^26^, the biological N fixation^23^, and the potential of fungal disease occurrence in the proceeding crop^40^. These factors could contribute to the differences seen in the RSA of this study, such as the increased number of seminal roots when sown after wheat compared to a rotational precrop like OSR, seen in both regions.

### RSA variability across cultivars and their interaction with the environment

Within this study, it can be seen that there is a genetic component to the RSA within the Swedish cultivar germplasm that causes differential RSA in the same conditions. This intra-species variation in RSA has been noted multiple times^41^ and can be linked with cultivar superiority in certain environmental conditions^42^. Our data shows that regional differences (such as soil composition), temporal and regional differences (such as weather patterns), and managerial differences (such as different precrops), interact with the genetic differences between cultivars, resulting in different RSA. Thus we can see that environmental conditions will affect this germplasm differently, causing different RSA traits.

Nodal and seminal roots have different internal structures that give them alternate properties that better suit diverse environments and conditions^43^. Seminal and nodal roots act differently to stress, based on numerous mechanisms^44^. Our results show cultivar-dependent effects in nodal root angle depending on the region and precrop. The majority of the difference was seen in Ceylon which exhibited shallower roots after a wheat precrop, during the flowering stage. Seminal root differences are primarily cultivar-specific, mainly attributed to Julius, which exhibits fewer roots with a narrower angle. Therefore we can see that differences in seminal roots are much more genetically driven, whereas the nodal root traits result in the G × E interaction. The differences in RSA based on the environment could be due to the cultivar-specific sensitivity to distinct stresses, due to other tolerance mechanisms. It has been seen that separate stresses will cause the angle to be affected differently^34^. Alternatively, these differences in RSA could be due to connected but unrelated variables, such as the relationship between seed size and seminal root number^19^. Together these could indicate that managerial practices that affect root angle and number, and thus tolerance/susceptibility to stress, are both region and cultivar-dependent, both in Sweden and likely globally.

### Links to grain yield variability

Previous research has clearly shown a positive effect of rotational cropping on crop yield^45^. The results presented here have shown that, in this situation, the number of seminal and nodal roots could be positively correlated with grain yield, in a precrop/growth stage-dependent manner. This is in contrast to the results by Xu, et al.^46^, which showed a negative correlation between root number and grain yield, however, it has been seen that root system size alternates correlation based on environment^47^, and thus this correlation could depend on the environment, such as seen here for seminal root number. The correlations seen here show grain yield to be correlated with an increased seminal root number when grown after a rotational precrop but correlated with an increased nodal root number for both during elongation. Fradgley, et al.^32^ showed no correlation between nodal root number and yield in wheat, however, this was collected at the dough development stage after flowering, supporting the lack of relation after elongation found in this study. Whilst other studies have shown that farm management practices have effects on the RSA, with links to yield suggested^48^, the results here suggest that this link to yield can occur only under certain practices. Exemplifying this is the results in nodal root angle which this study shows to be linked to yield in a growth stage and precrop dependent manner. Changes in nodal root angle are usually associated with the depletion of soil resources such as water and nutrients^49–51^, therefore this highly dependent correlation highlights the diversity of the environment and the importance of understanding the environment to better adapt the RSA. These may go some way toward understanding why precrop effects are not always reported as positive^52^. Whilst our results indicate a relationship between root number and angle with yield, it is difficult to speculate about a direct mechanistic link between the two traits, especially in the context of the precrop-dependent manner, as both are possibly linked to an unidentified factor or crop property that is driving the changes in both. The grain yield correlation is supported, in part, by comparing the root number differences in the varieties and between the regions, and how this relates to their yield in each treatment. Seminal root number was more varied between cultivars than any other variable. Julius, with the lowest number of seminal roots, was amongst the lowest yielders of the five lines when grown after a rotational precrop for all sites over both years; whilst Informer, with the highest number of seminal roots, produced the highest or second-highest yield. This supports the relation found between seminal roots and yield when grown after a rotational precrop and suggests that it is, in part, the number of seminal roots affecting the yield. This is in contrast to the findings in previous studies that showed negative correlations^12^. However, similar trends are seen under certain conditions such as drought^53^. Nodal root number showed a correlation with yield when sampled at the elongation phase, whilst this is not seen in the varieties, it is seen in the trials, where the plants grown in the Bjertop trial have significantly more roots at elongation than in Svalöv after wheat, with similar trends in the standardized yields. However, this difference in nodal root number is much more pronounced in the flowering stage of 2021, for which there is no observed correlation. Root number being correlated with grain yield during the elongation phase shows the importance of quick root growth in order to achieve higher yields. The correlation of seminal root number only after an OSR precrop could be due to the comparatively reduced nutrient pressure when grown after OSR^54^, which has different nutritional demands, favouring plants with increased seminal roots to take quick advantage of the increased nutrients available. Differences in biopores could also explain the link between yield and seminal root number, as seminal roots require easy penetration for more effective use^55^, although a recent study has not shown any difference in soil structure between the two precrops used in this study^56^. As there are differences in the soil characteristics and nutrient conditions between the regions, it stands to reason that these differences, and thus potential root architectural advantages may be heightened or nullified depending on the environment. Additionally, different crop varieties, with genetics producing varying root numbers, can further exploit the differing environments caused by precrop, potentially further heightening or nullifying the advantages conferred by increased root numbers.

The grain yield correlation is also supported in part by the ranking of varieties for nodal root angle, with the ranking of varieties by nodal root angle at the elongation stage inversely matching the ranking of varieties by standardized yield, but only after an OSR precrop. This supports the correlation seen between these two variables during elongation when grown after an OSR precrop. However, at the flowering stage following wheat, the correlation is not supported by variations in variety ranking. What does support this trend is that the only significant disparity in nodal root angle between sites occurs between the OSR and wheat precrop treatments in one of the locations (Bjertorp). This discrepancy corresponds to a narrowed yield gap between precrops in Bjertorp compared to the other location (Svalöv). Seminal roots are largely genetically influenced, and thus are affected by the varieties, whereas the nodal roots are largely environmentally influenced^57^, and thus are affected by the differences in the trial sites. This could explain the trends of seminal roots being seen in the varietal difference and the trends in nodal roots being seen in the environmental differences. A narrower nodal root angle at elongation correlating with yield only after an OSR precrop suggests that the root systems benefit from going deeper after this precrop, but not wheat. OSR crops tend to have shallower rooting systems, thus depleting more resources on the surface^58^. A deeper rooting system at an early stage may allow for access to untapped nutrients after an OSR precrop, but this would not be the case after a wheat precrop as the roots would go to similar levels and thus areas within reach at this stage would be equally depleted.

This suggests that managerial practices, such as rotational cropping, can alleviate or amplify stresses on the plant that alter yield, and alternate RSA will interact with these differently, with some resulting in better yield stability. This could be due to the preceding crop's root systems affecting soil conditions such as biopores, weed and pathogen burden, and resource availability which have been shown to increase yield in crops^26,28^. Different RSA could help the plant adapt to these changes with mechanisms such as nodal root spread giving greater access to limited nutrients and water, more seminal roots accessing nutrients quicker in the early growth stage to promote quick establishment, or root system architecture that promotes the growth of beneficial microbe/limits growth of pathogens–as RSA has been shown to affect the root exudates released and thus alter the microbial community^59^.

### Implications for management and breeding

The data from this study suggests that certain cultivars within the modern cultivar germplasm grown in Sweden will produce an RSA that is more adapted to specific regions of Sweden and with distinct managerial practices. This can be seen clearly in the data presented here where the wheat varieties Ceylon and Brons, which have the largest nodal root angles after an OSR precrop, also produced the largest yields when grown after an OSR precrop. More diverse global germplasm would likely contain RSA better suited for a wider range of regions globally. A better understanding of the rooting behaviour of different cultivars would thus help us select cultivars best suited for the environment and best positioned to benefit from the managerial practices. Identification of cultivars, and their associated markers, that produce RSAs favourable for different regions will allow for the breeding, through marker-assisted selection, of specific RSA to tailor crops such as Swedish winter wheat to particular regions and practices^32^. This diversity in cultivars could help address the stagnation in wheat yield improvement seen in Europe and globally^60^. Additionally, the diversity of cultivars could increase crop stability in the face of climate change^61^, similar to, and in conjunction with, farm-type diversity^62^.

Further information about how the RSA affects the interaction with nutrients and microbial communities is needed, to understand what part of the different interaction between regions, precrops, and cultivars is caused by the root structure changing access to nutrients and colonisation of microbes (including prevention of pathogenicity). Increasing the number of regions, and the difference in the soil composition at these regions is also necessary to aid cultivar tailoring of RSA to a given region.

Our findings show that precrop has an impact on RSA, and this effect could play a role in the overall effects of precrop on crop performance and yield seen in previous studies^24–27^. Further assessment of the carry-over effects of precrop will also help us understand the changes they make to the soils^63^, and how this affects cultivar selection. Understanding the effect of the precrop on the soil environment, and how this interacts with the genetic element will allow an understanding of the mechanistic approaches that help adapt these cultivars to different conditions. The identification of these mechanisms, and the markers associated with them, will allow for the implementation of these root characteristics into breeding programs to tailor the genetic component to maximise yield stability in a given environment (both soil conditions and managerial practices, such as precrop) both in Sweden and globally.

### Future assessment

The key findings from this study are that there can be large effects of farm management practices, such as precrop, on the RSA; and that these effects can be altered by the environment in which the crop is grown. Further, these changes in RSA can be partly correlated with yield, with different traits affecting yield depending on practices. The variability in RSA found between the modern cultivars can also be seen in the interaction with this dynamic precrop effect, thus suggesting that some cultivars will have RSA better suited for growth in particular environments and conditions.

From this data, we are able to form hypotheses about the roles of root system architecture, with supporting evidence. We hypothesise that regional differences will alter the way that the RSA adapts to the precrop effect, thus showing regional-specific precrop effects. This is shown in the results here, indicating a wider nodal root angle with an OSR precrop in Svalöv but narrower in Bjertorp. This could be due to aspects such as soil properties causing/alleviating different stresses. Additionally, we know that the alternate root types play important roles at different growth stages and fulfil different roles, so we hypothesise that this contributes to part of the differences in both cultivar suitability to the environment and the differences in yield after different precrops. In this study, we see ample evidence that the two root types measured react differently to the type of preceding crop, with different aspects correlating on yield in a growth stage- and precrop-dependant manner. These hypotheses need further testing across a range of precrop scenarios, using more diverse material, and testing several different RSA traits.

## Conclusions

Current crop selection has been indirectly preferencing RSAs that reduce the crops’ ability to adapt to certain situations. The outcome of this selection is slower root growth and overall smaller root systems, limiting the crop’s ability to resist drought and use resources efficiently^11,32^. However, our results show that, in the modern cultivars on the Swedish market, there are still RSA differences that could potentially be utilised to increase yield stability in different environments and with different farm management practices. Specifically, this paper shows that there is a region-specific interaction with the preceding crop which affects the RSA and correlates to yield, with nodal root angle correlating with yield positively at the flowering stage when grown after a wheat precrop, but negatively at the elongation phase when grown after an OSR precrop. Understanding the mechanisms for these differences and how they interact could allow for the breeding of cultivars better adapted for localised regions around the world, concerning environment and practices, through high-throughput methods such as marker-assisted selection. This could help improve the yield and yield stability, as well as help climate change-proof the agricultural system by providing a diversity of cultivars and thus increasing the chances of having cultivars that can thrive in the unpredictable environments created by the changing climate.

## Materials and methods

### Study site

Field trials were part of a larger experiment, laid out by Lantmännen over two harvest years (2020 & 2021) as part of a larger study. This study included 25 Swedish cultivars each, which differed per year, and over four location sites in Sweden. Sampling was done mainly in two locations in Sweden to represent two regions: Svalöv (Skåne County, south) and Bjertorp (Västra Götaland County, west-centre). Each region comprises two field sites each year. These regions were geographically distinct, with different weather patterns and soil types. However, there will also be slight variation within a region that cannot be taken into account statistically. The sites in each region over all years were in close proximity of each other: within an area of 3.4 km^2^ with a minimum spanning tree (MST) distance of 6.2 km in Svalöv, and 4.3 km^2^ with an MST distance of 5.8 km in Bjertorp, calculated using the package GeoRange in R^64^. The distance between the two sites in each region, within a given year, is between 1.6–2.8 for Svalöv and 3.1–3.8 km for Bjertorp.

### Weather differences

Weather data was taken at both sites, showing increased temperature, humidity, accumulated precipitation, and reduced wind in Svalöv, compared to Bjertorp, for almost every monthly average of both years^65^(Supplementary Fig. 5). Differences can also be seen between years with small differences in precipitation in the second year, but much drier to much wetter extremes in Svalöv and Bjertorp, respectively, in the first year along with a much warmer winter in both sites.

### Soil differences

At each location, there were two field sites (a total of four sites per year); one that had winter wheat as a precrop (control), and the other winter oilseed rape (OSR). In Bjertorp the soils range from light to intermediate clay (20–35%), the pH ranges from 6–6.6, and the Phosphorus (P) and Potassium (K) classes are 2 and 3, respectively, for all fields (with the exception of the P class in the first year after OSR, which was 3). In Svalöv the soils were also intermediate clay (approximately 35–40% for all), with higher pH ranges of 6.4–7.5. The K class was similar to Bjertorp at 3 (with the first year after wheat at 4), but the P class was much higher than Bjertorp overall at 4 in the first year, with large differences in the second year 3 and 5 after wheat and OSR, respectively.

### Plant material

At each location site with the winter wheat as a precrop, there were 3 replicate plots of each cultivar grown. The sites with OSR had 2 replicates. The nitrogen application was approximately 200 kg N ha^−1^, varying depending on regional requirements.

Five cultivars were selected (Brons, Ceylon, Chevignon, Informer, and Julius), which were used in all field trials in both years, and plants (8 in 2020, and 5 in 2021) were taken from each 12 × 2 m plot. The samples were taken from the centre of the final 1 × 2 m section of each plot from both sites during the flowering stage in 2020. In 2021 samples were taken during both the late tillering/early stem elongation stage and flowering stage.

Additional cultivars were sampled in both years. These were cultivars Reform (in 2020), and GGT Saki, Hallfreda, and Kask (in 2021). As they were only taken from one year each they were not used in the main assessment, and only used in the regression and principle component analysis (PCA).

The plants were taken during the flowering stage using the shovelomics method that removed a section of soil approximately 15 cm on either side of the wheat row and 25 cm deep, taking the core of the root system. The plants were then kept cool while transported and kept in a 6.5 °C room. After ripening the plots were harvested recording the grain moisture, protein, starch, and yield (plot, and standard—adjusted to 14% moisture for standardized yield).

### Root architectural measurements

Excess soil was removed from the plant roots by allowing them to soak in water for 30 min and then gently rubbing the soil away. The roots were then washed with running water to remove any remaining soil, and excess water was removed. The number, and the angle between the outermost roots, of both the seminal and nodal roots, were measured for each plant. Nodal root angle was measured by gently shaking the roots so they hang naturally, and then recording the angles left and right of the centre using the Maize Shovelomics Scoreboard (Roots Lab, Penn State University, USA), and taking this away from 180 to find the total angle. The nodal root number was taken by counting and removing each root. This was repeated on the seminal roots to get the seminal root angle and number.

### Statistical analysis

Analysis was done for the 2 years combined for each of the root architectural variates measured, using only the two regions and five cultivars common to both. This was analysed with a mixed-effects model, using the statistical program ‘R’^66^, using packages lme4^67^, lmerTest^68^, pbkrtest^69^, and emmeans^70^. The fixed-effects factors were region, precrop, cultivar, and sampling—which was divided into three groups: 2020 sampling (flowering stage), first 2021 sampling (elongation stage), and second 2021 sampling (flowering stage). The structural factors were region: location: precrop:rep—with location being the specific location of the field within the region, which differed depending on precrop and sampling date. The data from the individual plants from each experimental unit was averaged to avoid pseudoreplication. Due to the heteroscedasticity of the models (Supplementary Fig. 6) for nodal root number this data set was transformed by taking the square root and re-modelled. An ANOVA was performed on this model, using Kenward-Roger’s method for denominator degrees-of-freedom.

Spearman’s and Pearson’s rank correlations were then performed between each of the variates, and between the variates and the traits measured during the harvesting, using ‘R’. This was done overall, but also when grouped by region, precrop, trial, and cultivar; using all data points. The PCA was done by first splitting the data into the different growth stages (Elongation and Flowering), These two datasets then had each variable standardized for each of the datasets, to keep the variance comparable within each sampling, and then splitting it based on the preceding crop (OSR and wheat). PCAs were then done on each of the four datasets created, using the seminal and nodal root numbers and angles, shoot number and dry weight, and the standardized yield. Visualisation of the analysed data was done using ‘R’ packages ggplot2^71^, ggpubr^72^, and rmisc^73^.

### Supplementary Information

Supplementary Information.

## Data Availability

Data is available on request from the corresponding author(s).
