# Supplementary material for: Clear effects on root system architecture of winter wheat cultivars (Triticum aestivum L.) from cultivation environment and practices
Source: Sci Rep. 2024 May 15;14:11099. doi: 10.1038/s41598-024-61765-1 (PMC11096180; doi:10.1038/s41598-024-61765-1)
Supplement: Supplementary file 1 — Supplementary Information. [file 41598_2024_61765_MOESM1_ESM.docx]

# Supplementary Material


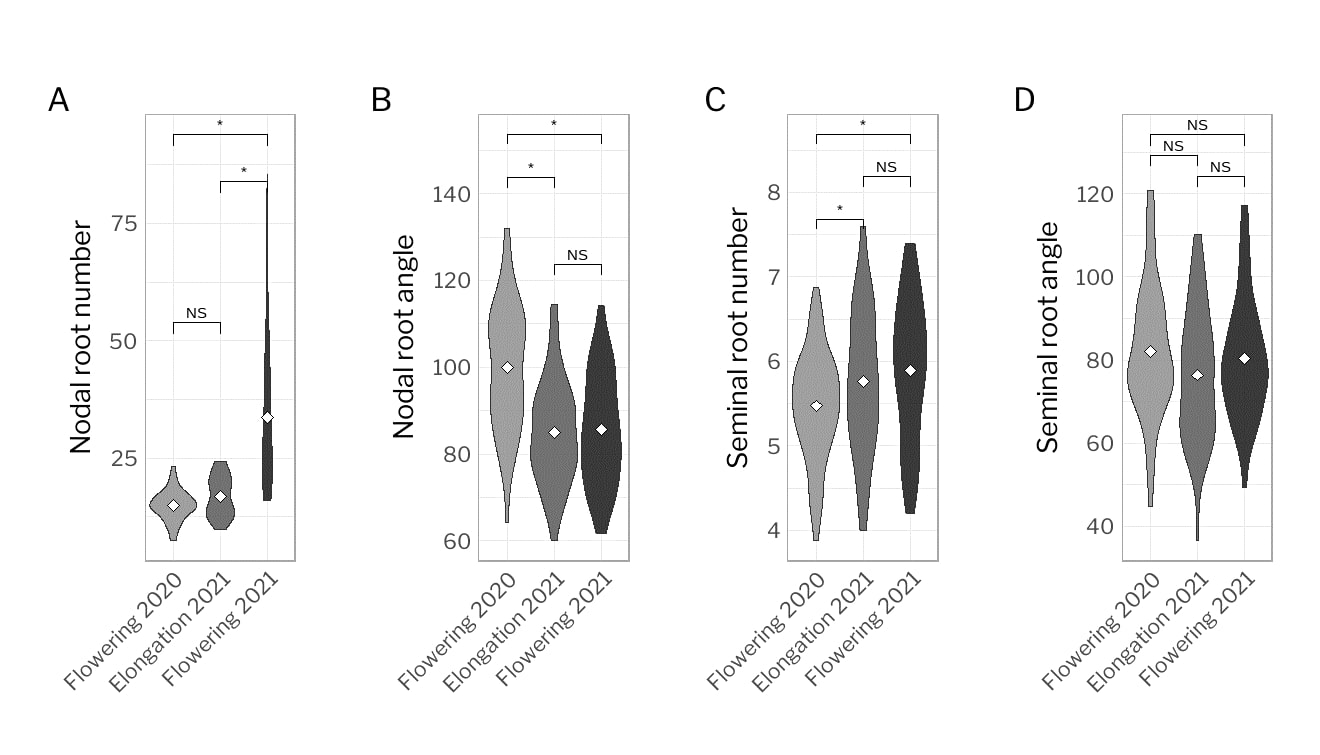


Supplementary Figure 1) Distribution of nodal root number (**A**) and angle (**B**), and seminal root number (**C**) and angle (**D**) between the three time periods over two growing seasons – Flowering in 2020, as well as Elongation and Flowering in 2021 – for wheat plants field-grown in two growing regions in Central and Southern Sweden. The significance is based on the estimated marginal means from the emmeans package in ‘R’ ^[73]^; denotations are ‘*’ for p < 0.05, and ‘NS’ for no significance.


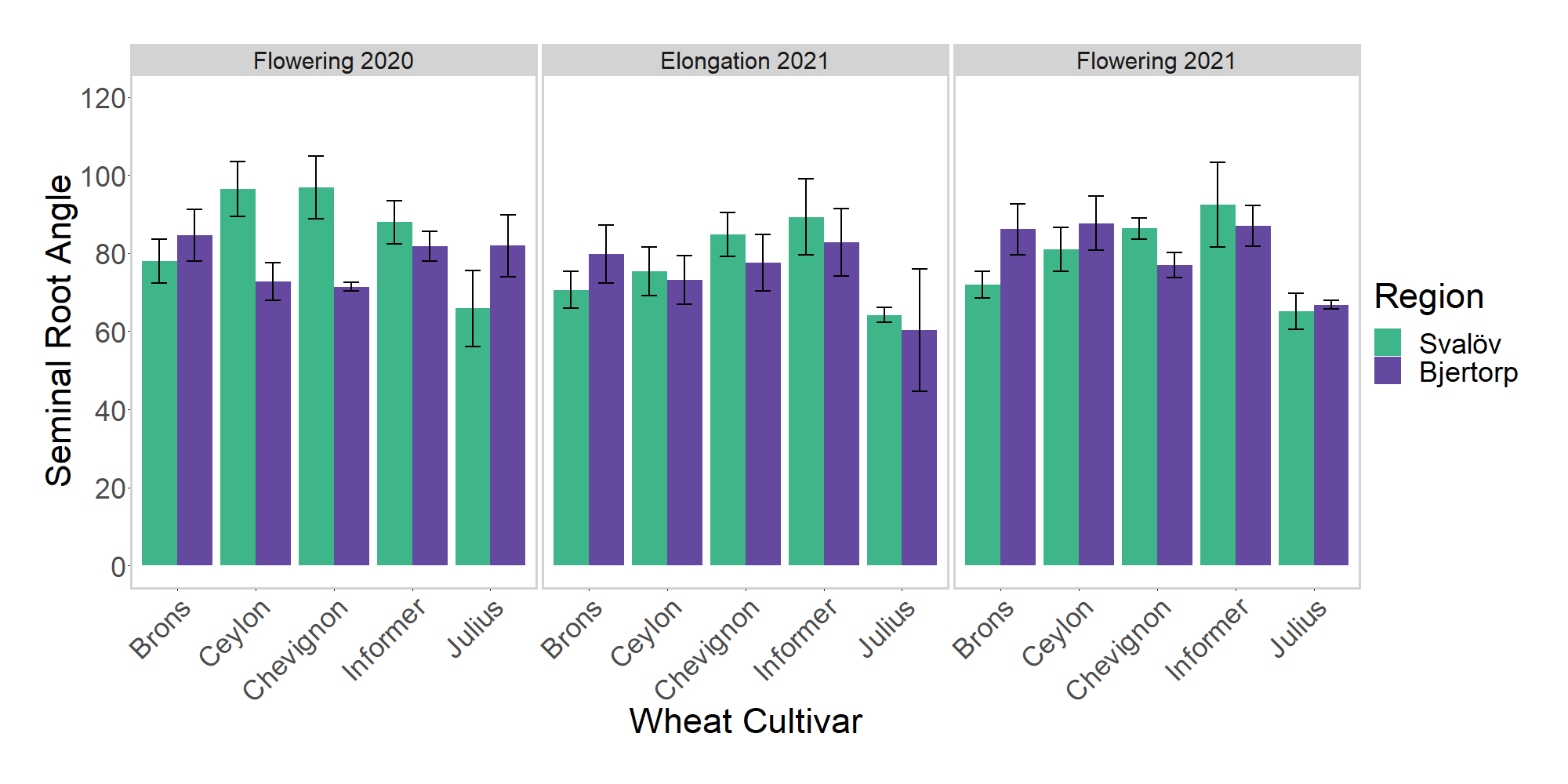


Supplementary Figure 2) Difference in seminal root angle between cultivars of wheat plants field-grown with two different preceding crops (OSR and Wheat). The data is divided by sampling period, and coloured depending on region – Svalöv in Southern Sweden (green) or Bjertorp in Central Sweden (purple).


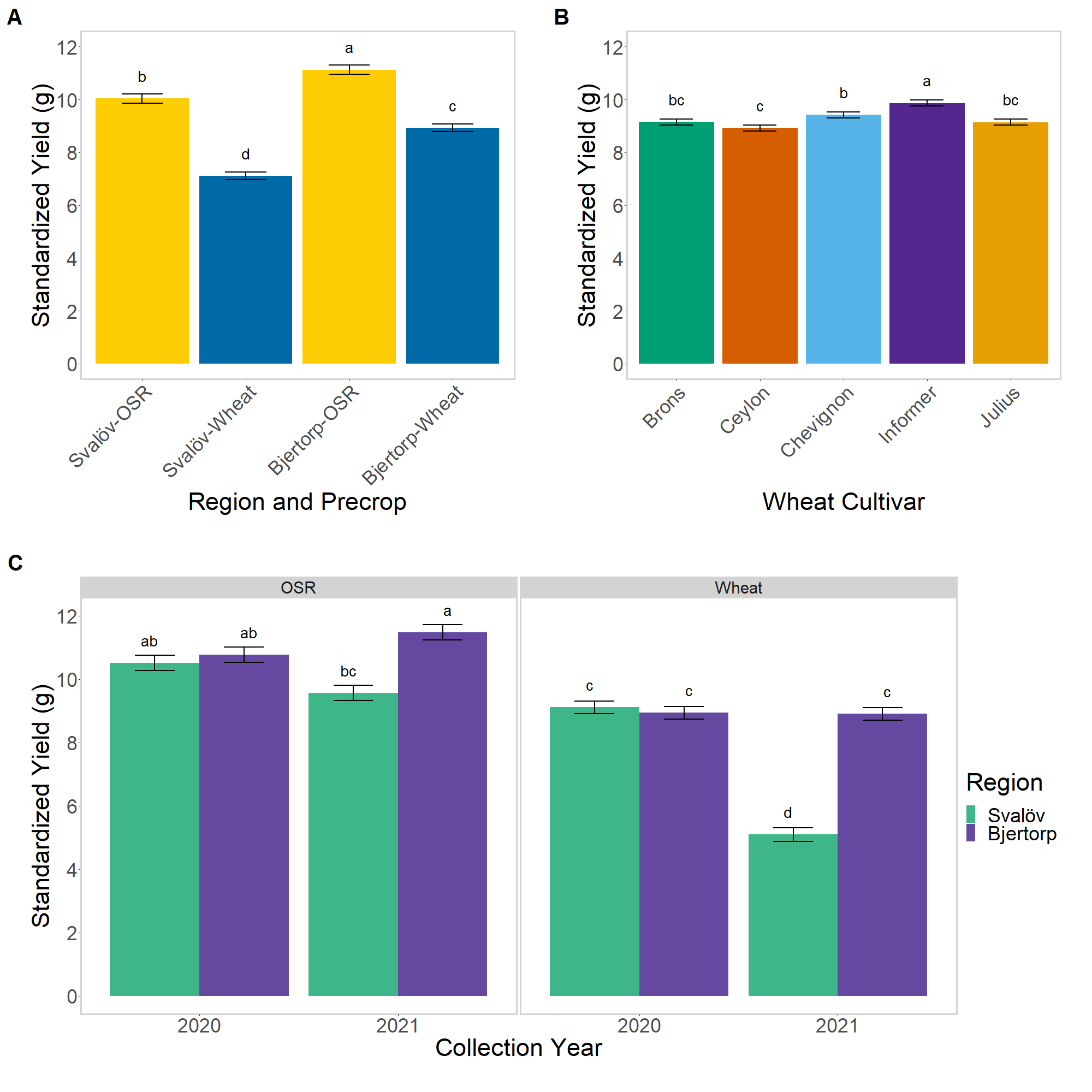


Supplementary Figure 3) Differences in standardised yield (yield standardized to a set moisture level) between field sites (combination of region and precrop; **A**), cultivars (**B**), or year – divided by precrop and coloured by region (**C**).


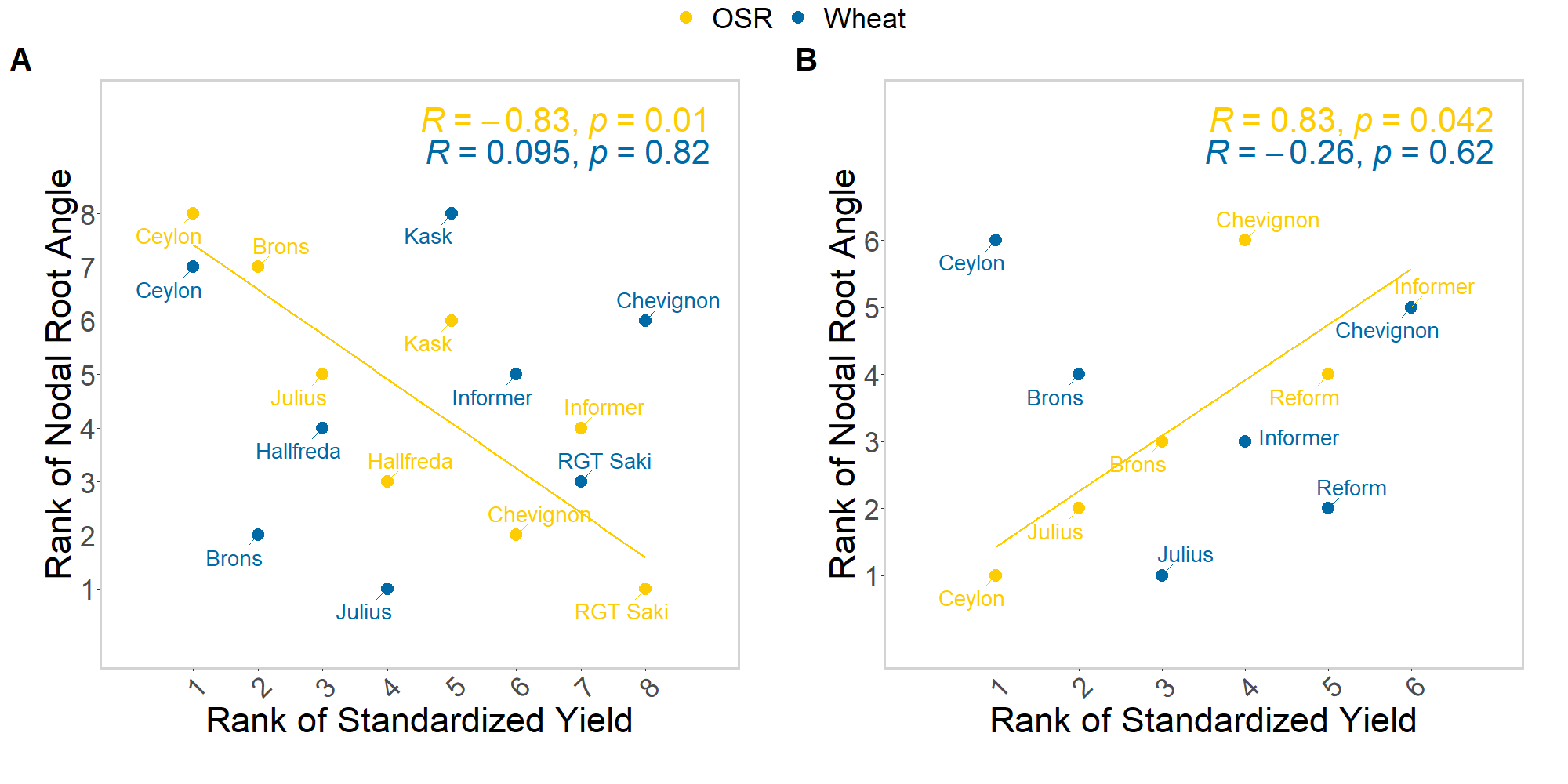


Supplementary Figure 4) Rank correlations (Pearson’s) of the wheat varieties (labelled) for yield values, standardized to account for differing moisture levels, vs nodal root angle at both the elongation (2021; **A**) and flowering (2020 & 2021; **B**) stage of wheat plants field-grown in two growing regions in Central and Southern Sweden. Split based on the precrop of either OSR (yellow) or wheat (blue) with regression lines fitter when significant.

Supplementary Figure 5) Weather data, from regions Svalöv and Bjertorp, for the two growing seasons using the average for the months, for temperature (**A**), humidity (**B**), and windspeed (**D**), as well as the accumulated precipitation for the cultivation period by month (**C**). Data was taken from ^[68]^. The locations of the sites in Sweden are marked on the map (**E**) with the region coloured for Svalöv (Skåne; green) and Bjertorp (Västergötland; purple), made using SimpleMappr (https://www.simplemappr.net/).


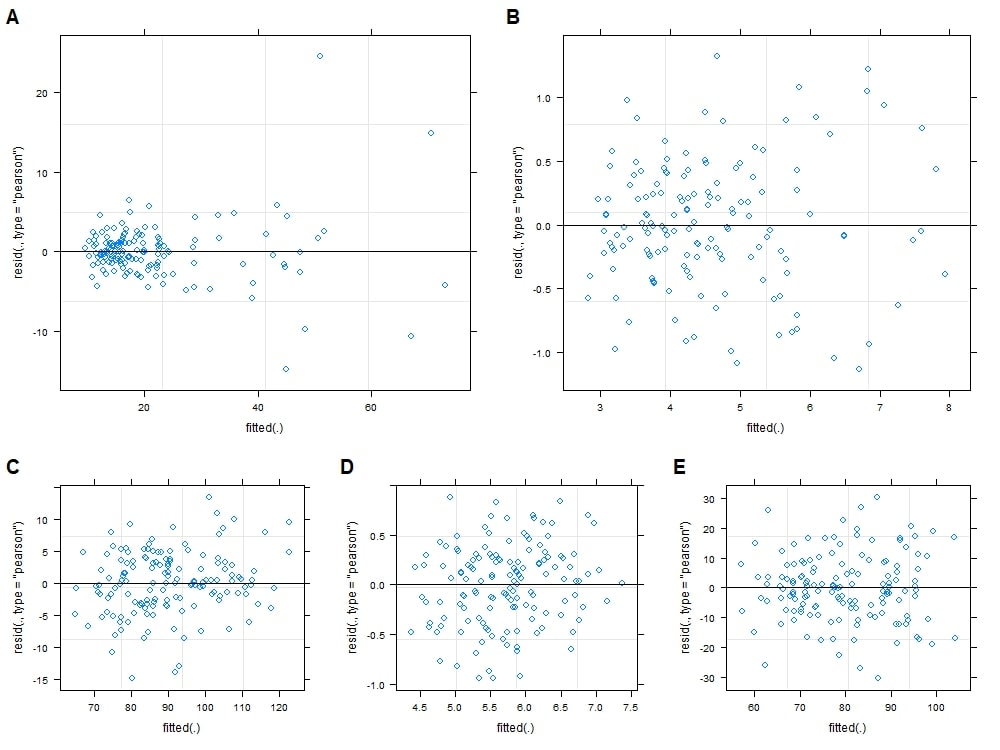


Supplementary Figure 6) Plots of the mixed-effect models to check for heteroscedasticity for the variables; nodal root number – untransformed (**A**) and square root transformed (**B**), nodal root angle (**C**), seminal root number (**D**), and seminal root angle (**E**).


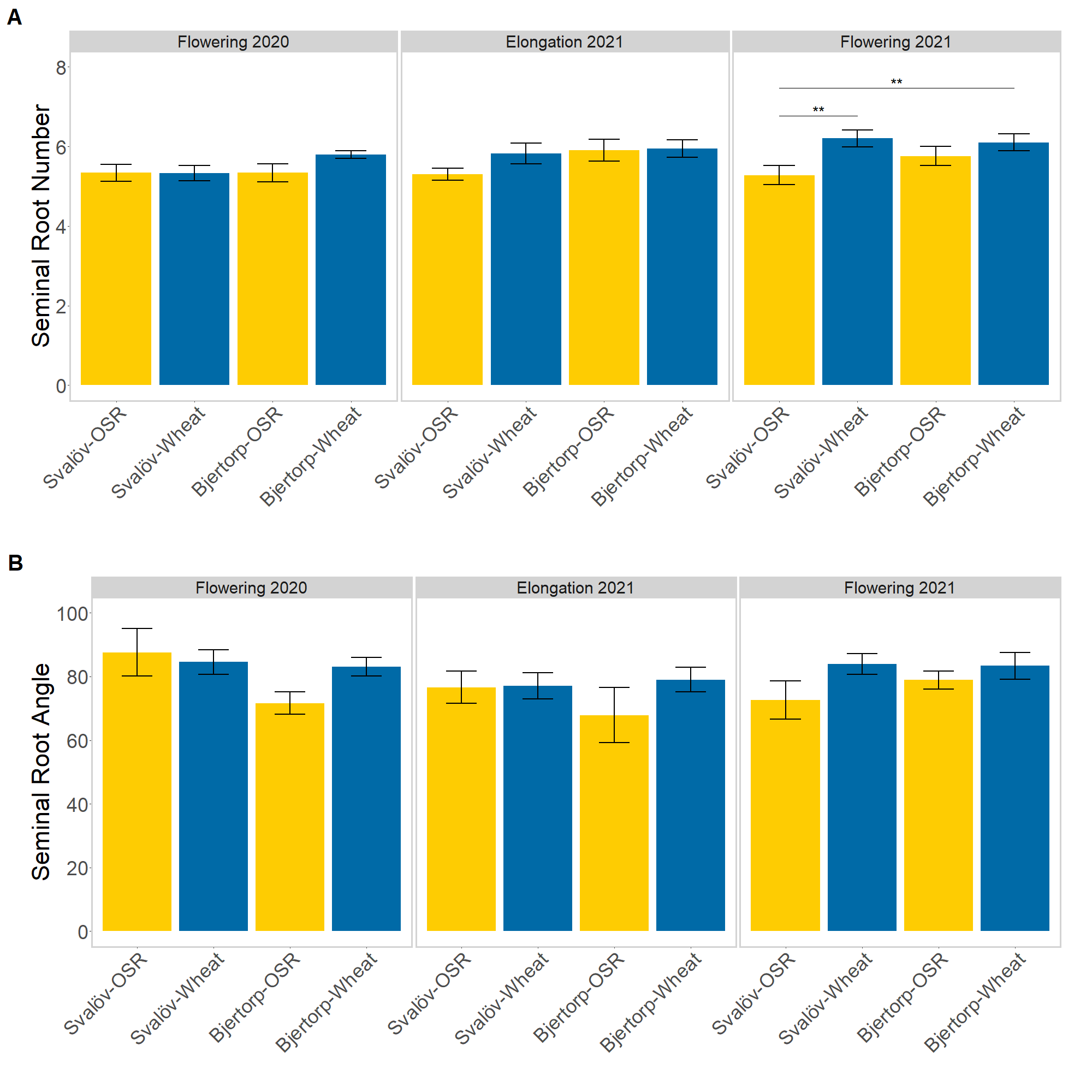


Supplementary Figure 7) Differences in seminal root number (**A**) and angle (**B**), between the four different field sites for wheat plants field-grown in two growing regions in Central (Bjertorp) and Southern Sweden (Svalöv) with two field sites with different precrops in each region – OSR (yellow) and wheat (Blue), and faceted by sampling time. The significant difference is only within the same facet and is based on the estimated marginal means from the emmeans package in ‘R’ ^[65]^; denotations are ‘*’ for p < 0.05, ‘*’ for p < 0.01, and ‘***’ for p < 0.0001.

Supplementary Table 1) Summary of the significance (displaying only the significant comparisons) based on the estimated marginal means from the emmeans package in ‘R’ ^[73]^, for nodal root number and angle grown on four different field sites for wheat plants field-grown in two growing regions in Central (Bjertorp) and Southern Sweden (Svalöv) with two field sites with different precrops in each region. Comparing the precrop – OSR and wheat, sampling period, and cultivar tested.

| Contrast | Nodal Root Number p-value | | Nodal Root Angle p-value |
| --- | --- | --- | --- |
| Elongation 2021 Brons Good - Flowering 2021 Julius Good | | 0.004 | 1.000 |
| Elongation 2021 Brons Good - Flowering 2021 Brons Wheat | | <.0001 | 1.000 |
| Elongation 2021 Brons Good - Flowering 2020 Ceylon Wheat | | 1.000 | 0.009 |
| Elongation 2021 Brons Good - Flowering 2021 Ceylon Wheat | | 0.040 | 1.000 |
| Elongation 2021 Brons Good - Flowering 2021 Informer Wheat | | <.0001 | 1.000 |
| Elongation 2021 Brons Good - Flowering 2021 Julius Wheat | | 0.038 | 1.000 |
| Flowering 2020 Brons Good - Flowering 2021 Brons Good | | 0.040 | 1.000 |
| Flowering 2020 Brons Good - Flowering 2021 Ceylon Good | | 0.047 | 0.756 |
| Flowering 2020 Brons Good - Flowering 2021 Julius Good | | 0.001 | 1.000 |
| Flowering 2020 Brons Good - Flowering 2021 Brons Wheat | | <.0001 | 1.000 |
| Flowering 2020 Brons Good - Flowering 2021 Ceylon Wheat | | 0.004 | 1.000 |
| Flowering 2020 Brons Good - Flowering 2021 Informer Wheat | | <.0001 | 1.000 |
| Flowering 2020 Brons Good - Flowering 2021 Julius Wheat | | 0.004 | 1.000 |
| Flowering 2021 Brons Good - Elongation 2021 Ceylon Good | | 0.021 | 1.000 |
| Flowering 2021 Brons Good - Flowering 2020 Ceylon Good | | 0.002 | 1.000 |
| Flowering 2021 Brons Good - Flowering 2020 Chevignon Good | | 0.030 | 0.076 |
| Flowering 2021 Brons Good - Elongation 2021 Informer Good | | 0.009 | 1.000 |
| Flowering 2021 Brons Good - Flowering 2020 Informer Good | | 0.017 | 0.848 |
| Flowering 2021 Brons Good - Elongation 2021 Julius Good | | 0.004 | 1.000 |
| Flowering 2021 Brons Good - Flowering 2020 Julius Good | | 0.006 | 1.000 |
| Flowering 2021 Brons Good - Elongation 2021 Brons Wheat | | 0.007 | 1.000 |
| Flowering 2021 Brons Good - Flowering 2020 Brons Wheat | | 0.005 | 0.216 |
| Flowering 2021 Brons Good - Elongation 2021 Ceylon Wheat | | 0.005 | 0.999 |
| Flowering 2021 Brons Good - Flowering 2020 Ceylon Wheat | | 0.005 | 0.001 |
| Flowering 2021 Brons Good - Elongation 2021 Chevignon Wheat | | 0.022 | 1.000 |
| Flowering 2021 Brons Good - Flowering 2020 Chevignon Wheat | | 0.011 | 0.043 |
| Flowering 2021 Brons Good - Elongation 2021 Informer Wheat | | 0.010 | 1.000 |
| Flowering 2021 Brons Good - Flowering 2020 Informer Wheat | | <.0001 | 0.977 |
| Flowering 2021 Brons Good - Elongation 2021 Julius Wheat | | 0.007 | 1.000 |
| Flowering 2021 Brons Good - Flowering 2020 Julius Wheat | | <.0001 | 1.000 |
| Elongation 2021 Ceylon Good - Flowering 2021 Ceylon Good | | 0.045 | 0.374 |
| Elongation 2021 Ceylon Good - Flowering 2021 Julius Good | | <.0001 | 1.000 |
| Elongation 2021 Ceylon Good - Flowering 2021 Brons Wheat | | <.0001 | 1.000 |
| Elongation 2021 Ceylon Good - Flowering 2021 Ceylon Wheat | | 0.006 | 1.000 |
| Elongation 2021 Ceylon Good - Flowering 2021 Informer Wheat | | <.0001 | 1.000 |
| Elongation 2021 Ceylon Good - Flowering 2021 Julius Wheat | | 0.006 | 1.000 |
| Flowering 2020 Ceylon Good - Flowering 2021 Ceylon Good | | 0.002 | 0.991 |
| Flowering 2020 Ceylon Good - Flowering 2021 Julius Good | | <.0001 | 1.000 |
| Flowering 2020 Ceylon Good - Flowering 2021 Brons Wheat | | <.0001 | 1.000 |
| Flowering 2020 Ceylon Good - Flowering 2020 Ceylon Wheat | | 1.000 | 0.014 |
| Flowering 2020 Ceylon Good - Flowering 2021 Ceylon Wheat | | <.0001 | 1.000 |
| Flowering 2020 Ceylon Good - Flowering 2021 Chevignon Wheat | | 0.004 | 1.000 |
| Flowering 2020 Ceylon Good - Flowering 2021 Informer Wheat | | <.0001 | 1.000 |
| Flowering 2020 Ceylon Good - Flowering 2021 Julius Wheat | | <.0001 | 1.000 |
| Flowering 2021 Ceylon Good - Flowering 2020 Chevignon Good | | 0.035 | 0.002 |
| Flowering 2021 Ceylon Good - Elongation 2021 Informer Good | | 0.011 | 1.000 |
| Flowering 2021 Ceylon Good - Flowering 2020 Informer Good | | 0.020 | 0.169 |
| Flowering 2021 Ceylon Good - Elongation 2021 Julius Good | | 0.005 | 1.000 |
| Flowering 2021 Ceylon Good - Flowering 2020 Julius Good | | 0.007 | 0.999 |
| Flowering 2021 Ceylon Good - Elongation 2021 Brons Wheat | | 0.008 | 1.000 |
| Flowering 2021 Ceylon Good - Flowering 2020 Brons Wheat | | 0.006 | 0.007 |
| Flowering 2021 Ceylon Good - Elongation 2021 Ceylon Wheat | | 0.006 | 0.599 |
| Flowering 2021 Ceylon Good - Flowering 2020 Ceylon Wheat | | 0.006 | <.0001 |
| Flowering 2021 Ceylon Good - Elongation 2021 Chevignon Wheat | | 0.027 | 0.882 |
| Flowering 2021 Ceylon Good - Flowering 2020 Chevignon Wheat | | 0.013 | 0.001 |
| Flowering 2021 Ceylon Good - Elongation 2021 Informer Wheat | | 0.012 | 0.992 |
| Flowering 2021 Ceylon Good - Flowering 2020 Informer Wheat | | 0.001 | 0.325 |
| Flowering 2021 Ceylon Good - Elongation 2021 Julius Wheat | | 0.009 | 1.000 |
| Flowering 2021 Ceylon Good - Flowering 2020 Julius Wheat | | <.0001 | 0.919 |
| Elongation 2021 Chevignon Good - Flowering 2020 Chevignon Good | | 1.000 | 0.007 |
| Elongation 2021 Chevignon Good - Flowering 2021 Julius Good | | 0.003 | 1.000 |
| Elongation 2021 Chevignon Good - Flowering 2020 Brons Wheat | | 1.000 | 0.022 |
| Elongation 2021 Chevignon Good - Flowering 2021 Brons Wheat | | <.0001 | 0.999 |
| Elongation 2021 Chevignon Good - Flowering 2020 Ceylon Wheat | | 1.000 | <.0001 |
| Elongation 2021 Chevignon Good - Flowering 2021 Ceylon Wheat | | 0.035 | 0.978 |
| Elongation 2021 Chevignon Good - Flowering 2020 Chevignon Wheat | | 1.000 | 0.003 |
| Elongation 2021 Chevignon Good - Flowering 2021 Informer Wheat | | <.0001 | 0.991 |
| Elongation 2021 Chevignon Good - Flowering 2021 Julius Wheat | | 0.034 | 1.000 |
| Flowering 2020 Chevignon Good - Elongation 2021 Informer Good | | 1.000 | 0.041 |
| Flowering 2020 Chevignon Good - Flowering 2021 Julius Good | | 0.001 | 0.123 |
| Flowering 2020 Chevignon Good - Elongation 2021 Brons Wheat | | 1.000 | 0.004 |
| Flowering 2020 Chevignon Good - Flowering 2021 Brons Wheat | | <.0001 | 0.212 |
| Flowering 2020 Chevignon Good - Flowering 2021 Ceylon Wheat | | 0.003 | 0.423 |
| Flowering 2020 Chevignon Good - Flowering 2021 Chevignon Wheat | | 0.078 | 0.047 |
| Flowering 2020 Chevignon Good - Flowering 2021 Informer Wheat | | <.0001 | 0.331 |
| Flowering 2020 Chevignon Good - Elongation 2021 Julius Wheat | | 1.000 | 0.002 |
| Flowering 2020 Chevignon Good - Flowering 2021 Julius Wheat | | 0.003 | 0.148 |
| Flowering 2021 Chevignon Good - Flowering 2020 Ceylon Wheat | | 0.176 | 0.001 |
| Flowering 2021 Chevignon Good - Flowering 2020 Chevignon Wheat | | 0.300 | 0.041 |
| Flowering 2021 Chevignon Good - Flowering 2020 Informer Wheat | | 0.030 | 0.975 |
| Flowering 2021 Chevignon Good - Flowering 2021 Informer Wheat | | 0.004 | 1.000 |
| Flowering 2021 Chevignon Good - Flowering 2020 Julius Wheat | | 0.002 | 1.000 |
| Elongation 2021 Informer Good - Flowering 2021 Julius Good | | <.0001 | 1.000 |
| Elongation 2021 Informer Good - Flowering 2021 Brons Wheat | | <.0001 | 1.000 |
| Elongation 2021 Informer Good - Flowering 2020 Ceylon Wheat | | 1.000 | <.0001 |
| Elongation 2021 Informer Good - Flowering 2021 Ceylon Wheat | | 0.002 | 1.000 |
| Elongation 2021 Informer Good - Flowering 2020 Chevignon Wheat | | 1.000 | 0.020 |
| Elongation 2021 Informer Good - Flowering 2021 Informer Wheat | | <.0001 | 1.000 |
| Elongation 2021 Informer Good - Flowering 2021 Julius Wheat | | 0.002 | 1.000 |
| Flowering 2020 Informer Good - Flowering 2021 Julius Good | | 0.001 | 0.926 |
| Flowering 2020 Informer Good - Flowering 2021 Brons Wheat | | <.0001 | 0.991 |
| Flowering 2020 Informer Good - Flowering 2021 Ceylon Wheat | | 0.001 | 1.000 |
| Flowering 2020 Informer Good - Flowering 2021 Chevignon Wheat | | 0.044 | 0.825 |
| Flowering 2020 Informer Good - Flowering 2021 Informer Wheat | | <.0001 | 0.999 |
| Flowering 2020 Informer Good - Flowering 2021 Julius Wheat | | 0.001 | 0.964 |
| Flowering 2021 Informer Good - Flowering 2021 Informer Wheat | | 0.002 | 1.000 |
| Flowering 2021 Informer Good - Flowering 2020 Julius Wheat | | 0.004 | 1.000 |
| Elongation 2021 Julius Good - Flowering 2021 Julius Good | | 0.001 | 1.000 |
| Elongation 2021 Julius Good - Flowering 2021 Brons Wheat | | <.0001 | 1.000 |
| Elongation 2021 Julius Good - Flowering 2020 Ceylon Wheat | | 1.000 | 0.001 |
| Elongation 2021 Julius Good - Flowering 2021 Ceylon Wheat | | 0.001 | 1.000 |
| Elongation 2021 Julius Good - Flowering 2020 Chevignon Wheat | | 1.000 | 0.030 |
| Elongation 2021 Julius Good - Flowering 2021 Chevignon Wheat | | 0.036 | 1.000 |
| Elongation 2021 Julius Good - Flowering 2021 Informer Wheat | | <.0001 | 1.000 |
| Elongation 2021 Julius Good - Flowering 2021 Julius Wheat | | 0.001 | 1.000 |
| Flowering 2020 Julius Good - Flowering 2021 Julius Good | | <.0001 | 1.000 |
| Flowering 2020 Julius Good - Flowering 2021 Brons Wheat | | <.0001 | 1.000 |
| Flowering 2020 Julius Good - Flowering 2020 Ceylon Wheat | | 1.000 | 0.005 |
| Flowering 2020 Julius Good - Flowering 2021 Ceylon Wheat | | <.0001 | 1.000 |
| Flowering 2020 Julius Good - Flowering 2021 Chevignon Wheat | | 0.016 | 1.000 |
| Flowering 2020 Julius Good - Flowering 2021 Informer Wheat | | <.0001 | 1.000 |
| Flowering 2020 Julius Good - Flowering 2021 Julius Wheat | | <.0001 | 1.000 |
| Flowering 2021 Julius Good - Elongation 2021 Brons Wheat | | <.0001 | 1.000 |
| Flowering 2021 Julius Good - Flowering 2020 Brons Wheat | | <.0001 | 0.326 |
| Flowering 2021 Julius Good - Elongation 2021 Ceylon Wheat | | <.0001 | 1.000 |
| Flowering 2021 Julius Good - Flowering 2020 Ceylon Wheat | | <.0001 | 0.002 |
| Flowering 2021 Julius Good - Elongation 2021 Chevignon Wheat | | 0.001 | 1.000 |
| Flowering 2021 Julius Good - Flowering 2020 Chevignon Wheat | | <.0001 | 0.075 |
| Flowering 2021 Julius Good - Elongation 2021 Informer Wheat | | <.0001 | 1.000 |
| Flowering 2021 Julius Good - Flowering 2020 Informer Wheat | | <.0001 | 0.995 |
| Flowering 2021 Julius Good - Elongation 2021 Julius Wheat | | <.0001 | 1.000 |
| Flowering 2021 Julius Good - Flowering 2020 Julius Wheat | | <.0001 | 1.000 |
| Elongation 2021 Brons Wheat - Flowering 2020 Brons Wheat | | 1.000 | 0.013 |
| Elongation 2021 Brons Wheat - Flowering 2021 Brons Wheat | | <.0001 | 0.978 |
| Elongation 2021 Brons Wheat - Flowering 2020 Ceylon Wheat | | 1.000 | <.0001 |
| Elongation 2021 Brons Wheat - Flowering 2021 Ceylon Wheat | | <.0001 | 0.985 |
| Elongation 2021 Brons Wheat - Flowering 2020 Chevignon Wheat | | 1.000 | 0.001 |
| Elongation 2021 Brons Wheat - Flowering 2021 Chevignon Wheat | | 0.005 | 1.000 |
| Elongation 2021 Brons Wheat - Flowering 2021 Informer Wheat | | <.0001 | 0.995 |
| Elongation 2021 Brons Wheat - Flowering 2021 Julius Wheat | | <.0001 | 1.000 |
| Flowering 2020 Brons Wheat - Flowering 2021 Brons Wheat | | <.0001 | 0.511 |
| Flowering 2020 Brons Wheat - Flowering 2021 Ceylon Wheat | | <.0001 | 0.793 |
| Flowering 2020 Brons Wheat - Flowering 2021 Chevignon Wheat | | 0.012 | 0.140 |
| Flowering 2020 Brons Wheat - Flowering 2021 Informer Wheat | | <.0001 | 0.691 |
| Flowering 2020 Brons Wheat - Elongation 2021 Julius Wheat | | 1.000 | 0.004 |
| Flowering 2020 Brons Wheat - Flowering 2021 Julius Wheat | | <.0001 | 0.386 |
| Flowering 2021 Brons Wheat - Elongation 2021 Ceylon Wheat | | <.0001 | 1.000 |
| Flowering 2021 Brons Wheat - Flowering 2020 Ceylon Wheat | | <.0001 | 0.002 |
| Flowering 2021 Brons Wheat - Elongation 2021 Chevignon Wheat | | <.0001 | 1.000 |
| Flowering 2021 Brons Wheat - Flowering 2020 Chevignon Wheat | | <.0001 | 0.125 |
| Flowering 2021 Brons Wheat - Elongation 2021 Informer Wheat | | <.0001 | 1.000 |
| Flowering 2021 Brons Wheat - Flowering 2020 Informer Wheat | | <.0001 | 1.000 |
| Flowering 2021 Brons Wheat - Elongation 2021 Julius Wheat | | <.0001 | 0.990 |
| Flowering 2021 Brons Wheat - Flowering 2020 Julius Wheat | | <.0001 | 1.000 |
| Elongation 2021 Ceylon Wheat - Flowering 2020 Ceylon Wheat | | 1.000 | 0.038 |
| Elongation 2021 Ceylon Wheat - Flowering 2021 Ceylon Wheat | | <.0001 | 1.000 |
| Elongation 2021 Ceylon Wheat - Flowering 2021 Chevignon Wheat | | 0.003 | 0.999 |
| Elongation 2021 Ceylon Wheat - Flowering 2021 Informer Wheat | | <.0001 | 1.000 |
| Elongation 2021 Ceylon Wheat - Flowering 2021 Julius Wheat | | <.0001 | 1.000 |
| Flowering 2020 Ceylon Wheat - Flowering 2021 Ceylon Wheat | | <.0001 | 0.008 |
| Flowering 2020 Ceylon Wheat - Elongation 2021 Chevignon Wheat | | 1.000 | 0.008 |
| Flowering 2020 Ceylon Wheat - Flowering 2021 Chevignon Wheat | | 0.011 | <.0001 |
| Flowering 2020 Ceylon Wheat - Elongation 2021 Informer Wheat | | 1.000 | 0.004 |
| Flowering 2020 Ceylon Wheat - Flowering 2021 Informer Wheat | | <.0001 | 0.004 |
| Flowering 2020 Ceylon Wheat - Elongation 2021 Julius Wheat | | 1.000 | <.0001 |
| Flowering 2020 Ceylon Wheat - Flowering 2020 Julius Wheat | | 0.993 | 0.020 |
| Flowering 2020 Ceylon Wheat - Flowering 2021 Julius Wheat | | <.0001 | 0.001 |
| Flowering 2021 Ceylon Wheat - Elongation 2021 Chevignon Wheat | | <.0001 | 1.000 |
| Flowering 2021 Ceylon Wheat - Flowering 2020 Chevignon Wheat | | <.0001 | 0.304 |
| Flowering 2021 Ceylon Wheat - Elongation 2021 Informer Wheat | | <.0001 | 1.000 |
| Flowering 2021 Ceylon Wheat - Flowering 2020 Informer Wheat | | <.0001 | 1.000 |
| Flowering 2021 Ceylon Wheat - Elongation 2021 Julius Wheat | | <.0001 | 0.910 |
| Flowering 2021 Ceylon Wheat - Flowering 2020 Julius Wheat | | <.0001 | 1.000 |
| Elongation 2021 Chevignon Wheat - Flowering 2021 Chevignon Wheat | | 0.039 | 1.000 |
| Elongation 2021 Chevignon Wheat - Flowering 2021 Informer Wheat | | <.0001 | 1.000 |
| Elongation 2021 Chevignon Wheat - Flowering 2021 Julius Wheat | | <.0001 | 1.000 |
| Flowering 2020 Chevignon Wheat - Flowering 2021 Chevignon Wheat | | 0.028 | 0.019 |
| Flowering 2020 Chevignon Wheat - Flowering 2021 Informer Wheat | | <.0001 | 0.221 |
| Flowering 2020 Chevignon Wheat - Elongation 2021 Julius Wheat | | 1.000 | <.0001 |
| Flowering 2020 Chevignon Wheat - Flowering 2021 Julius Wheat | | 0.001 | 0.086 |
| Flowering 2021 Chevignon Wheat - Elongation 2021 Informer Wheat | | 0.010 | 1.000 |
| Flowering 2021 Chevignon Wheat - Flowering 2020 Informer Wheat | | 0.001 | 0.974 |
| Flowering 2021 Chevignon Wheat - Flowering 2021 Informer Wheat | | 0.001 | 1.000 |
| Flowering 2021 Chevignon Wheat - Elongation 2021 Julius Wheat | | 0.005 | 1.000 |
| Flowering 2021 Chevignon Wheat - Flowering 2020 Julius Wheat | | <.0001 | 1.000 |
| Elongation 2021 Informer Wheat - Flowering 2021 Informer Wheat | | <.0001 | 1.000 |
| Elongation 2021 Informer Wheat - Flowering 2021 Julius Wheat | | <.0001 | 1.000 |
| Flowering 2020 Informer Wheat - Flowering 2021 Informer Wheat | | <.0001 | 1.000 |
| Flowering 2020 Informer Wheat - Flowering 2021 Julius Wheat | | <.0001 | 0.999 |
| Flowering 2021 Informer Wheat - Elongation 2021 Julius Wheat | | <.0001 | 0.956 |
| Flowering 2021 Informer Wheat - Flowering 2020 Julius Wheat | | <.0001 | 1.000 |
| Elongation 2021 Julius Wheat - Flowering 2021 Julius Wheat | | <.0001 | 0.993 |
| Flowering 2020 Julius Wheat - Flowering 2021 Julius Wheat | | <.0001 | 1.000 |
